# Room‐Temperature Transport Properties of Graphene with Defects Derived from Oxo‐Graphene

**DOI:** 10.1002/chem.201905252

**Published:** 2020-02-03

**Authors:** Zhenping Wang, Qirong Yao, Siegfried Eigler

**Affiliations:** ^1^ Institute for Chemistry and Biochemistry Freie Universität Berlin Takustraße 3 14195 Berlin Germany; ^2^ Physics of Interfaces and Nanomaterial University of Twente Enschede 7500 AE The Netherlands

**Keywords:** defects, electronic transport, graphene, graphene oxide

## Abstract

In recent years, graphene oxide has been considered as a soluble precursor of graphene for electronic applications. However, the performance lags behind that of graphene due to lattice defects. Here, the relation between the density of defects in the range of 0.2 % and 1.5 % and the transport properties is quantitatively studied. Therefore, the related flakes of monolayers of graphene were prepared from oxo‐functionalized graphene (oxo‐G). The morphologic structure of oxo‐G was imaged by atomic force microscopy (AFM) and scanning tunneling microscopy (STM). Field‐effect mobility values were determined to range between 0.3 cm^2^ V^−1^ s^−1^ and 33.2 cm^2^ V^−1^ s^−1^, which were inversely proportional to the density of defects. These results provide the first quantitative description of the density of defects and transport properties, which plays an important role for potential applications.

## Graphical abstract

**The effect of defects**: The room‐temperature mobility values of single‐layer graphene derived from oxo‐graphene with densities of defects between 0.2 % and 1.5 % are presented. The fundamental findings can explain the generally diverging results reported for reduced graphene oxide used in applications.

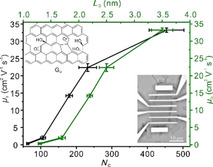

Chemically modified graphene, such as graphene oxide (GO) or oxo‐functionalized graphene (oxo‐G), has received considerable interests for electronic,^1^ optoelectronic,^2^ biological^3^ and chemical sensing^4^ applications due to its physicochemical phenomena, including its tunable bandgap,^5^ diverse luminescence behaviors,^6^ biological compatibility^7^ and the ability to modify the surface covalently and non‐covalently.^8^ In contrast to pristine graphene with carbon atoms arranged into a two‐dimensional hexagonal lattice, oxo‐G consists of abundant sp^3^‐hybridized carbon atoms, which are covalently bound to oxo‐groups, mainly hydroxyl and epoxy groups.^9^ The sp^3^‐portion is between 4 % and 60 %, with variable functionality.^10^ The oxo‐addends are tentatively immobilized onto segregated carbon, which isolates intact nanometer‐scale graphene domains into small islands.^11^ The existence of surface oxo‐groups has profound impacts on improving its hydrophilicity,^12^ chemical reactivity,^13^ catalytic activity,^14^ and optical properties,2a whereas the effect is detrimental for the electrical conductivity.^15^ Therefore, to enhance the electrical performance of GO or oxo‐G, extensive researches were conducted on the deoxygenation of oxo‐addends.^16^ In this regard, thermal processing provides a simple and versatile method for carbonization, however, without a carbon source, more lattice defects, such as few‐atoms vacancies and nanometer‐scale holes, are introduced due to thermal disproportionation along with CO_2_ formation.^17^ During the oxidative synthesis of GO and oxo‐G, defects are introduced into the carbon framework.10a They cannot be healed out by simple chemical reduction although oxo‐addends are reductively defunctionalized from the carbon lattice by a chemical reductant.^18^ The defect concentration in GO or oxo‐G can be determined by Raman spectroscopy after chemical reduction and typically varies from 0.001 % to 2 %.^19^

We demonstrated that the mobility of charge carriers of reduced oxo‐G with a density of defects as low as 0.02 % is exceeding 1000 cm^2^ V^−1^ s^−1^, measured at 1.6 K in a Hall bar configuration and about 200 cm^2^ V^−1^ s^−1^ for a density of defects of about 0.3 %.^20^ Those values are state‐of‐the art, but a series of questions arise. No systematic investigation is available relating the density of defects to the charge carrier mobilities at room temperature. Most reported values of carrier mobility values had been determined from multilayer thin films of reduced GO related materials with unknown thickness and qualities. Moreover, taking various synthetic procedures of oxo‐G and non‐standard transport measurements into consideration, there exist more uncertainties.

Here, we analyzed the structural evolution of single‐layer oxo‐G with defects from 0.2 % to 1.5 % by using AFM, STM and Raman spectroscopy. In addition, we investigated the effect of structural defects on the electrical properties of monolayer oxo‐G by two‐probe measurements under ambient conditions. Our results quantify the correspondence relationship between structural defects and transport capacities in high‐defect single‐layer graphene materials.

As shown in Figure 1, there are two types of graphene used in our investigation, almost defect‐free graphene as reference sample prepared through tape exfoliation,^21^ termed as G_0 %_ (index indicates the density of defects, *I*_D_/*I*_G_=0.2±0.07, which relates to a density of defects of 0.001 %, abbreviated G_0 %_) and graphene with various densities of defects (termed as G_D_ with density if defects between 0.2 % and up to 1.5 % determined by the relation to *I*_D_/*I*_G_ ratios, compare Figure S1, Supporting Information) obtained by wet‐chemical preparation and reduction with hydroiodic acid (HI).20a Surface morphologies of the samples were measured by atomic force microscopy (AFM) in tapping mode. As shown in Figure 2 a, the average height of G_0 %_ is determined to about 0.5 nm with lateral dimensions of 10–20 μm. Contaminants are visible at the edge of the G_0 %_ sheet, which stems from the used tape during the exfoliation and transfer processes. In contrast, the roughness of G_D_ is with 1.0 nm twice as high as for G_0 %_ due to bitopic oxo‐groups at the carbon basal plane and possible adsorbates (Figure 2 b). The lateral size of the depicted G_D_ flake was determined to be about 20 μm. To obtain information on the local atomic structure, scanning tunneling microscopy (STM) was used. Comparing the atomically‐resolved honeycomb structure for defect‐free highly ordered pyrolytic graphite (HOPG) in Figure 2 c, the single‐layer G_D_ reveals distinguishable topographical features with the appearance of islands and rows at bright spots, as depicted in Figure 2 d. The amorphous networks arise from the presence of defects in the carbon lattices, such as residual oxo‐groups, presumably at defect‐sites, vacancies and nm‐scale holes, as evidenced before.10b, 17a, 22

**Figure 1:**
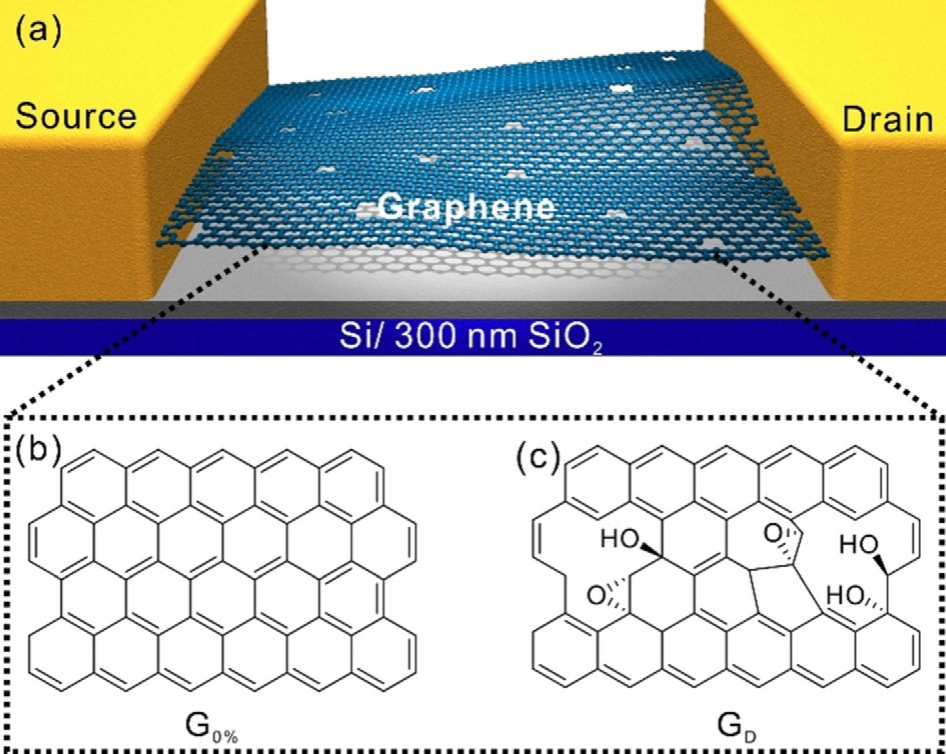
(a) Three‐dimensional schematic of a graphene‐based field‐effect transistor (FET). Schematic illustration of the chemical structure of (b) defect‐free graphene (G_0 %_) and (c) graphene with defects (G_D_).

**Figure 2:**
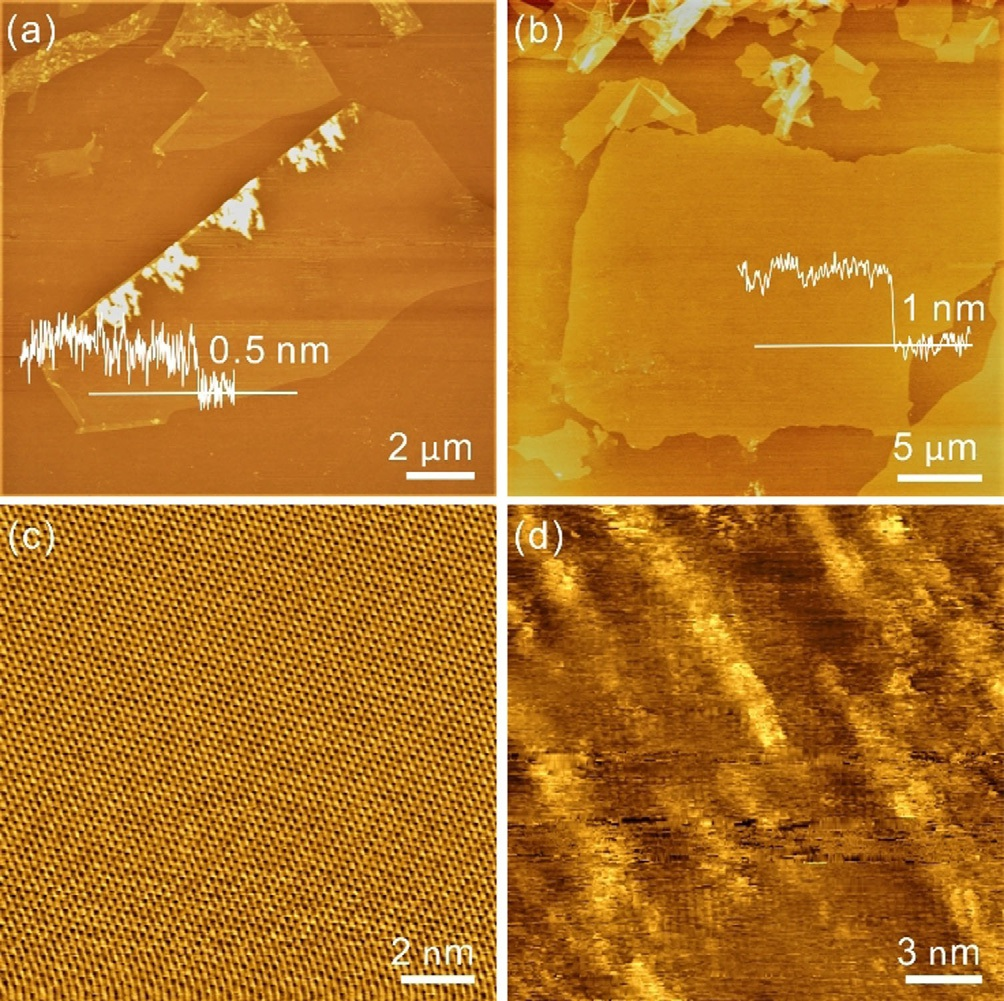
(a, b) AFM images of single‐layer G_0 %_ flake on a Si/SiO_2_ substrate and a single‐layer G_D_ flake on a Si/SiO_2_ substrate. (c, d) High‐resolution STM topographic images of HOPG and a single‐layer G_D_ flake on HOPG.

The density of defects in single layers of graphene can be quantified by Raman spectroscopy. Figure 3 a shows the evolution of Raman spectra obtained from single layers of graphene with various densities of defects. For the monolayer G_0 %_, there are two distinct peaks, the G band (at 1570 cm^−1^), associated with the in‐plane stretching vibration of C−C bonds, and the 2D band (at 2670 cm^−1^), activated by a double‐resonant Raman scattering.^23^ The G and 2D bands are sensitive to the structure of the carbon hexagonal lattice and, thus, they can be used to characterize the quality of graphene‐based materials. In addition, a faint D band at around 1360 cm^−1^ is noticed, probably evolving from grain boundaries or other lattice imperfections.^24^ The structural defects can be estimated by using the defect‐activated D band. Moreover, the intensity and shape of these peaks strongly depend on the nature of disorder. As the amount of disorder in graphene increases, the D‐band intensities enhance, whereas the 2D‐band intensities decrease. Further, the G‐band splits into two sub‐bands, G band (1583 cm^−1^) and D′ band (1620 cm^−1^). In addition, the broadening of all bands is observed with increasing density of defects. The full‐width‐at‐half‐maximum (FWHM, *Γ*) of the 2D peak (*Γ*_2D_) increases from approximately 24 cm^−1^ in the single‐layer G_0 %_ to about 178 cm^−1^ in the single‐layer G_1.5 %_. In Table 1, the details of Raman peak analyses are summarized for graphene samples with different densities of defects, namely G_0 %_, G_0.2 %_, G_0.4 %_, G_0.5 %_, G_0.9 %_ and G_1.5 %_.

**Figure 3:**
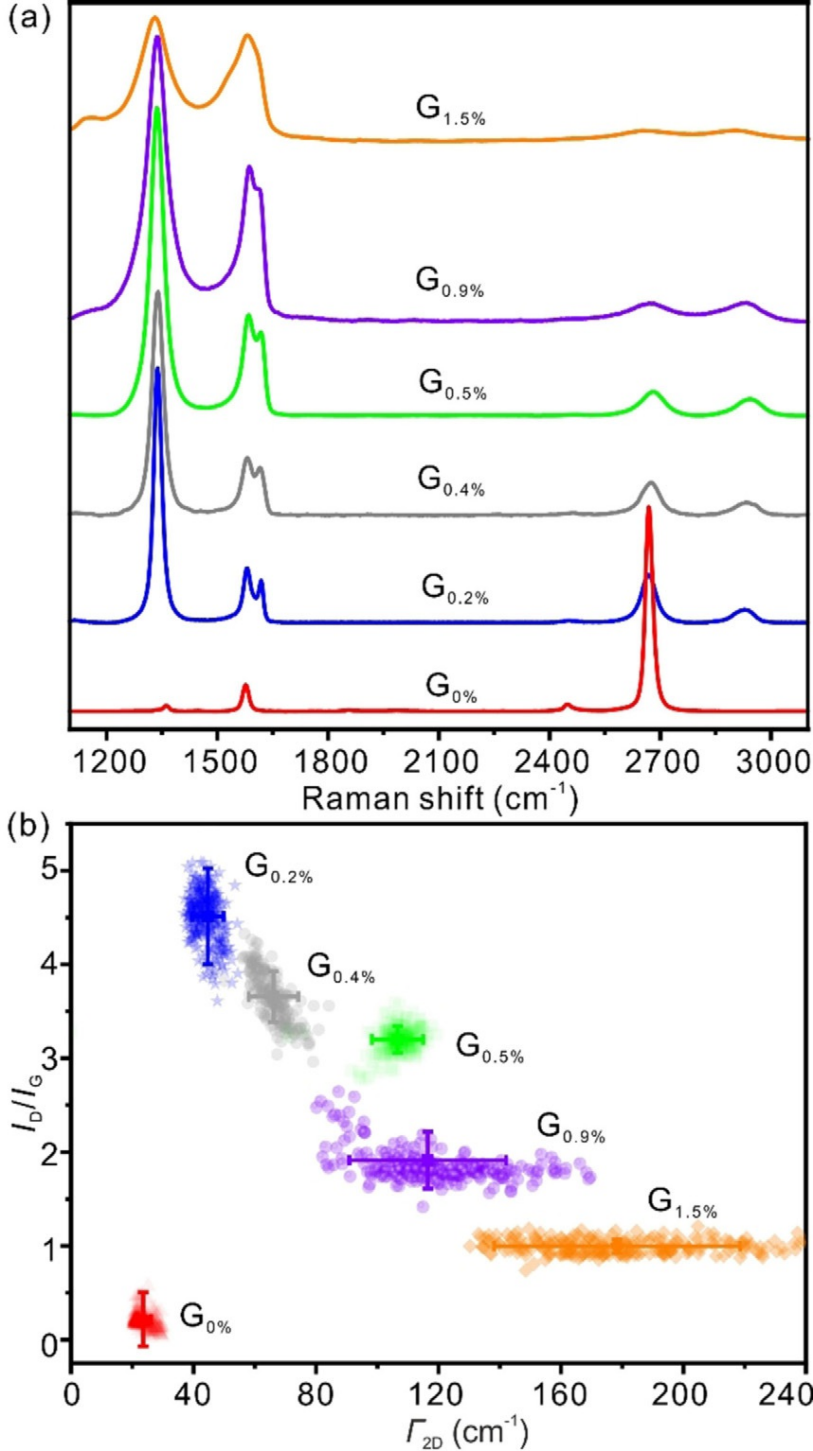
(a) Raman spectra obtained with a 532 nm excitation laser for single‐layer graphene with various densities of defects, namely G_0 %_, G_0.2 %_, G_0.4 %_, G_0.5 %_, G_0.9 %_ and G_1.5 %_. (b) Corresponding plot of the *I*_D_/*I*_G_ ratio vs. *Γ*_2D_.

**Table 1:** Summary of the results of fitting Raman spectra by Lorentz functions for the yielded monolayer graphene with defects densities of 0 %, 0.2 %, 0.4 %, 0.5 %, 0.9 % and 1.5 %.

Sample	*Γ*_D_ [cm^−1^]	*Γ*_G_ [cm^−1^]	*Γ*_2D_ [cm^−1^]	*I*_D_/*I*_G_	*N*_C_	*L*_D_ [nm]	*n*_D_ [cm^−2^]
G_0 %_	≈0	14.2±4.8	23.6±2.6	0.2±0.07	>24×10^3^	>25	≈5.1×10^10^
G_0.2 %_	25.2±3.0	40.5±8.5	44.7±5.1	4.5±0.5	454±49	3.5±0.19	≈2.6×10^12^
G_0.4 %_	38.1±2.9	41.3±3.1	66.1±8.1	3.7±0.2	232±25	2.5±0.13	≈5.1×10^12^
G_0.5 %_	46.3±3.9	33.7±3.0	106.9±5.6	3.2±0.1	180±9	2.2±0.05	≈6.6×10^12^
G_0.9 %_	67.5±6.4	58.5±27.1	118.6±18.6	1.9±0.2	107±7	1.7±0.06	≈1.1×10^13^
G_1.5 %_	91.0±5.7	120.3±42.4	178.3±40.1	1.0±0.1	63±3	1.3±0.03	≈1.9×10^13^

The intensity ratio of *I*_D_/*I*_G_ is used for determining the density of defects in the G_D_ samples. In the case of single‐layer G_0 %_, it contains an extremely low density of defects, which belongs to the low‐defect density regime according to the Raman model introduced by Lucchese, Cançado and Ferrari et al., with a ratio of *I*_D_/*I*_G_=0.2±0.07 corresponding to about 24 000 C atoms within the intact graphene lattice. According to Equation (1):

(1) $$N_{C}={2L}_{D}^{2}{2D}_{D}^{2}/A_{cell}$$

in which the *N*_C_ corresponds to intact carbon atoms and *A*_cell_=0.246^2^×sin (60°)=0.05239 nm^2^, the average distance between defects *L*_D_ is about 25 nm. The related defect density (*n*_D_) is about 5.1×10^10^ cm^−2^, using *n*_D_ (cm^−2^)=10^14^/(πL_D_^2^).19b However, the investigated G_D_ samples relate to the high‐defect density regime. In this regime, the *I*_D_/*I*_G_ ratio increases with an increase of *L*_D_, based on the relation of *I*_D_/*I*_G_∝*L*_D_∝*N*_C_.19b Accordingly, the density of defects increases from 0.3 % to 1.5 %, the corresponding *I*_D_/*I*_G_ ratio decreases from 4.5 to 1.0 and the *L*_D_ values gradually decrease from 3.5 nm to 1.3 nm, respectively. The related *n*_D_ increases from about 2.6×10^12^ cm^−2^ to 1.9×10^13^ cm^−2^. As depicted in Figure 3 b, the evolution of qualities in the yielded graphene samples are illustrated by plotting the *I*_D_/*I*_G_ ratio versus *Γ*_2D_. With increasing the density of defects, the *Γ*_2D_ values increase, which is consistent with our discussion above.

Field‐effect transistors were fabricated by using the obtained monolayer graphene samples as conducting channels (Figure 4 a). The monolayer G_0 %_ flakes were mechanically exfoliated from a bulk graphite and transferred to a heavily p‐doped Si substrate with a 300 nm thick SiO_2_ layer (Si/SiO_2_),^21^ which acts as the reference sample. The *Γ*_2D_ value of 23.6±2.6 and *I*_2D_/*I*_G_>4 was determined by Raman spectroscopy to prove the single‐layer nature of the produced flake (Figure 3 a). The monolayer G_D_ flakes prepared by wet‐chemistry were deposited on Si/SiO_2_ substrates by using Langmuir–Blodgett technique and subsequent chemical reduction or thermal processing.20a An AFM image of G_0.5 %_ FET device is shown in Figure 4 b and a height profile of monolayer G_0.5 %_ flake with about 1.2 nm is depicted in Figure 4 c.The Si/SiO_2_ substrate serves as a back‐gate. The metal contacts Cr/Au (5 nm/70 nm), served as source and drain electrodes, were deposited onto single‐layer graphene channel materials by using e‐beam lithography and evaporation processes. Avoiding any thermal decomposition of chemically‐derived G_D_ samples, no annealing process was performed for all prepared devices after the lift‐off process.

**Figure 4:**
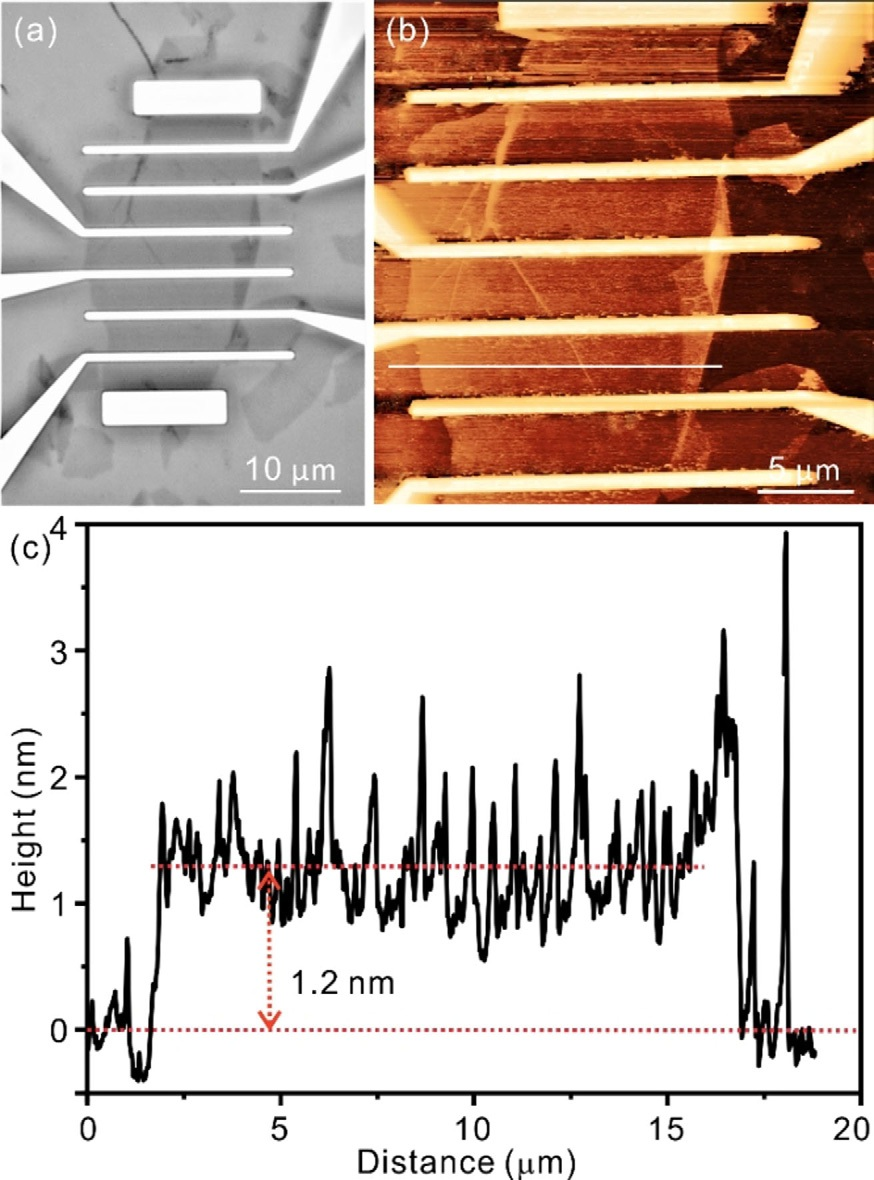
(a) Optical microscope image of G_0.5 %_ field‐effect transistor (FET). The monolayer G_0.5 %_ flake is the channel material. The Si/300 nm SiO_2_ substrate acts as a back‐gate. The Cr/Au (5 nm/70 nm) contacts are used for two‐probe connection. (b) AFM image of G_0.5 %_ FET device. (c) Height profile of the monolayer G_0.5 %_ flake along grey line shown in (b).

Electrical transport measurements were performed at ambient conditions in a two‐terminal configuration. The performance of transistors relies on the properties of channel materials, gate dielectrics, electrodes and test conditions. Therefore, to reliably compare electrical performances for the obtained monolayer graphene samples, all transistors were prepared with parallel electrodes, the same manufacturing processes and test conditions.

The Figure 5 presents the transfer characteristics of fabricated FET devices based on graphene samples with the defects from 0 % to 1.5 %. Linear output relations (*I*_DS_–*V*_DS_) are determined and visualized in the insets of Figure 5, indicating ohmic contacts between the graphene samples and the metal electrodes under ambient conditions. The G_0 %_ device in Figure 5 a shows V‐shape transfer curves (*I*_DS_–*V*_BG_) with asymmetric Dirac voltage (corresponding to the minimum value of *I*_DS_) located at +20 V. The observed p‐doping behavior was probably attributed to the heavily p‐doped Si/SiO_2_ substrate, impurity doping as a result of exfoliation and transfer processes or polar adsorbates like water or oxygen acting as charge traps between substrate and the graphene surface. Furthermore, a hysteretic behavior between forward and reverse sweeps are observed. For G_D_ transistors (Figure 5 b–f), no Dirac point appears within the range of the scanned gate voltages from −50 V to +50 V. All samples show unipolar p‐type character. These defective G_D_ samples are stronger p‐doped than the G_0 %_ sample due to the oxo‐groups modification of the graphene networks.^25^

**Figure 5:**
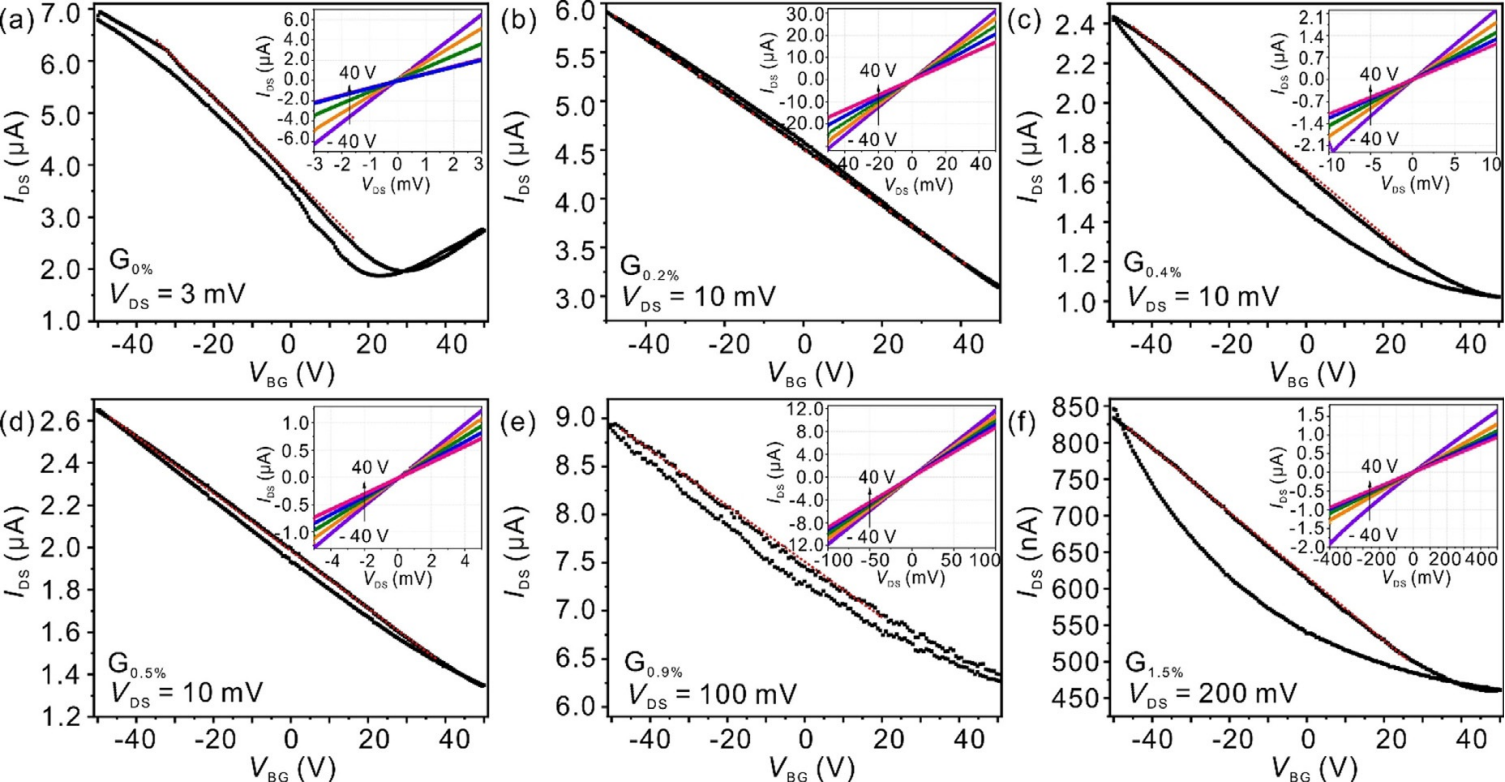
Room‐temperature transfer characteristics of graphene transistors with densities of defects of 0 %, 0.2 %, 0.4 %, 0.5 %, 0.9 % and 1.5 % (a–f), respectively. The gate voltage is swept continuously from −50 V to 50 V and back to −50 V. The inset shows the corresponding output curves.

The field‐effect carrier mobilities were extracted by using Equation (2):^26^

(2) $$\mu =\left(L/W\right)\times \left(1/C_{ox}V_{DS}\right)\times \left({dI}_{DS}/{dV}_{BG}\right)$$

in which *L* and *W* are the channel length and width, respectively, and *C*_ox_=1.15×10^−8^ F cm^−2^ is the capacitance per unit area of the gate dielectric material. The room‐temperature average mobility values of monolayers of G_D_ are determined between 33.2 cm^2^ V^−1^ s^−1^ and 0.3 cm^2^ V^−1^ s^−1^ for densities of defects between 0.2 % and 1.5 %. The mobility values are significantly lower than the value of 685 cm^2^ V^−1^ s^−1^ obtained for our defect‐free G_0 %_ and not annealed reference sample. In addition, in Figure 6 the field‐effect mobilities are plotted as a function of number of C atom (*N*_C_) and distance between defects (*L*_D_), respectively. It is found that the mobilities follow, within experimental uncertainties, a nonlinear relationship with *L*_D_ and *N*_C_, because the defect‐free area of graphene increases over‐proportionally with increasing *L*_D_.

**Figure 6:**
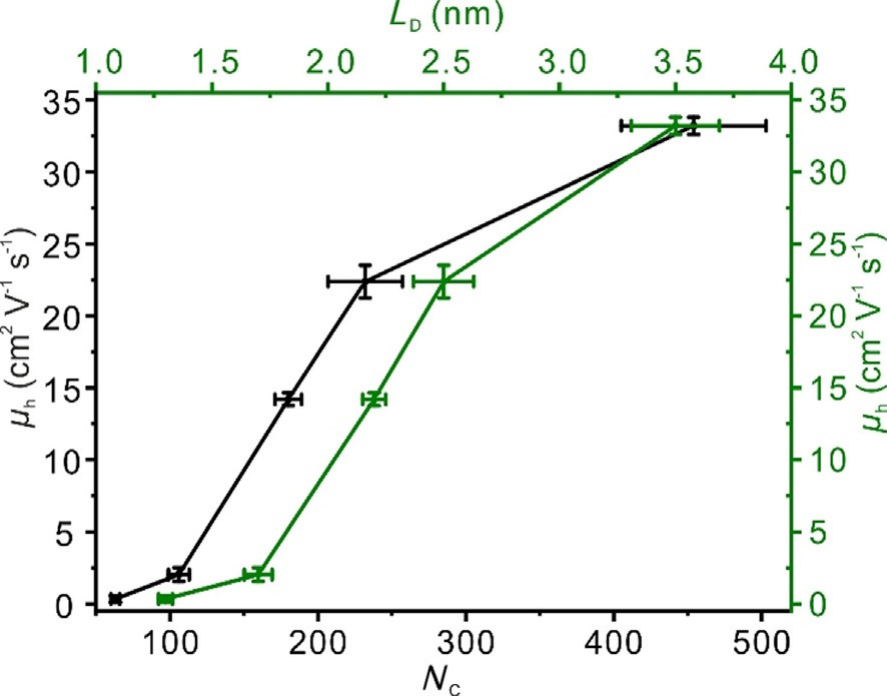
Field‐effect carrier mobility values as a function of number of C atoms (*N*_C_) of intact graphene areas (black curve) and the distance of defects *L*_D_ (green curve). The error bars shown are based on the data of Table 1 and Figure S2 (Supporting Information).

In summary, we have studied the room‐temperature electrical properties of single‐layer graphene derived from oxo‐G containing defect densities varying from 0.2 % to 1.5 %. The defects give rise to a heterogeneous topographical morphology of oxo‐G. The isolated graphene domains (*L*_D_≤3 nm) in oxo‐G were identified by Raman spectroscopy. The isolation of these domains limits the charge transport in reduced oxo‐G. Therefore, the mobility values of charge carriers of graphene with densities of defects between 0.2 % and 1.5 %, change by three orders of magnitude, from 0.3 cm^2^ V^−1^ s^−1^ and 33.2 cm^2^ V^−1s−1^. More generally, the mobility of charge carriers varies by orders of magnitude, although it looks like that the density of defects varies only a little. The fundamental findings reported here can explain the generally diverging results often reported for reduced graphene oxide used in applications.

## Experimental Section

### Methods

AFM characterization was performed by using a JPK NanoWizard 4 Atomic Force Microscope in tapping mode at room temperature. Raman characterization was carried out with a Horiba Explorer spectrometer with a 532 nm laser for excitation under air conditions. Scanning tunneling microscopy (STM) was conducted by using Omicron‐STM1 microscope under ultra‐high vacuum (<10^−10^ mbar). All transport measurements were performed under ambient conditions by a two‐probe station and two source‐measurement units (Keithley 2450).

### Preparation of defect‐free G_0 %_ flakes

The defect‐free monolayer G_0 %_ flakes were prepared by micromechanical exfoliation and then transferred on Si/SiO_2_ substrates as reported methods.^21^

### Preparation of defective G_D_ from oxo‐G

The defective G_D_ flakes were prepared by low‐temperature oxidation of graphite based by the before reported method of our group.20a Then, the oxo‐G was dissolved in methanol/water 1:1 mixtures. The monolayer flakes of G_D_ were deposited onto the Si/SiO_2_ substrate by using the Langmuir–Blodgett technique (LB, Kibron MicroTrough, 3 mN m^−1^ with the surface tension of water as reference value of 72.8 mN m^−1^). Reduction was performed by vapor of hydriodic acid (HI) and trifluoroacetic acid (TFA) (1:1, *v*/*v*) at 80 °C (10 min). Subsequently, the surface of G_D_ was cleaned with doubly distilled water (Carl Roth) to remove iodine species. The density of defects of individual flakes was determined by Raman spectroscopy (Horiba Explorer spectrometer with a 532 nm laser for excitation under air conditions). Subsequently, flakes with defined density of defects were selected for FET device fabrication.

### Fabrication of FET devices

Standard electron beam lithography procedure (Raith PIONEER TWO) was used to define and expose the geometry of metal contacts. Subsequently, a 5 nm/70 nm Cr/Au layer was deposited with thermal evaporation (Kurt J. Lesker NANO 36) and lifted off in acetone to make electrode contact to monolayer G_0 %_ and G_D_ flakes, respectively.

## Conflict of interest

The authors declare no conflict of interest.

## Supporting information

As a service to our authors and readers, this journal provides supporting information supplied by the authors. Such materials are peer reviewed and may be re‐organized for online delivery, but are not copy‐edited or typeset. Technical support issues arising from supporting information (other than missing files) should be addressed to the authors.

Supplementary
