# Supplementary material for: Room‐Temperature Transport Properties of Graphene with Defects Derived from Oxo‐Graphene
Source: Chemistry. 2020 Feb 3;26(29):6484–9. doi: 10.1002/chem.201905252 (PMC7317977; doi:10.1002/chem.201905252)
Supplement: Supplementary file 1 — Supplementary [file CHEM-26-6484-s001.pdf]

Chemistry–A European Journal

Supporting Information

Room-Temperature Transport Properties of Graphene with Defects Derived from Oxo-Graphene

Zhenping Wang,[a] Qirong Yao,[b] and Siegfried Eigler*[a]

1. Calculation of the defect density from Raman spectra

The calculation of the defect density is based on the model introduced and interpreted by Lucchese and Cançado and interpretation by Englert et al.[1] The detailed calculation processes are based on the following three equations.

$$v\frac{I_D}{I_G} = C_A \frac{r_A^2 - r_S^2}{r_A^2 - 2r_S^2}\left[e^{-\pi r_S^2 \omega / L_D^2} - e^{-\pi (r_A^2 - r_S^2) r_S^2 / L_D^2}\right] \quad (1)$$

$$C_A = (160 \pm 48) E_L^4 \quad (2)$$

$$N_C = 2L_D^2 / A_{cell} \quad (3)$$

Here the r_A and r_S are length scales that determine the region induced by the D band scattering. The C_A is related to the ratio between the efficiency of optical phonons between K and Γ. The E_L is the excitation energies (E_L = 2.33 eV, λ_L=532 nm). The N_C is the number of carbon atoms in graphene domains. The A_{cell} is a graphene unit cell, where the $A_{cell} = 0.246^2 \sin(60°) = 0.05239$ nm^2. The lines in Figure S1 are fitting curves following the three equations.

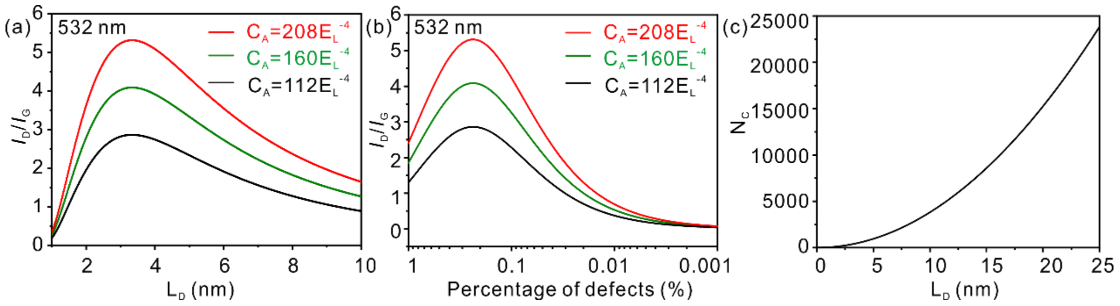

Figure S1. (a) I_D/I_G as s function of L_D. (b) I_D/I_G as s function of percentage of defects (%). The red, green and black curves are given by $C_A=208\times E_L^{-4}$, $C_A=160\times E_L^{-4}$ and $C_A=112\times E_L^{-4}$, respectively, where the excitation energies E_L of 532 nm laser is 2.33 eV. (c) The relation between N_C and L_D.

2. The resistance values measured by room-temperature two-probe configuration are summarized in the Table S1:

Table S1. Resistance values measured by room-temperature two-probe configuration are summarized.

Sample	$G_{0\%}$	$G_{0.2\%}$	$G_{0.4\%}$	$G_{0.5\%}$	$G_{0.9\%}$	$G_{1.5\%}$
resistance	0.9 kΩ	2.0 kΩ	7.9 kΩ	5.1 kΩ	131.7 kΩ	434.1 kΩ
Channel length	1.5 µm	2 µm	4 µm	2 µm	4 µm	5 µm

3. Room-temperature transfer characteristics of graphene transistors with densities of defects of 0%, 0.2%, 0.4%, 0.5%, 0.9% and 1.5%, respectively. The gate voltage is swept continuously from -50 V to 50 V and back to -50 V.

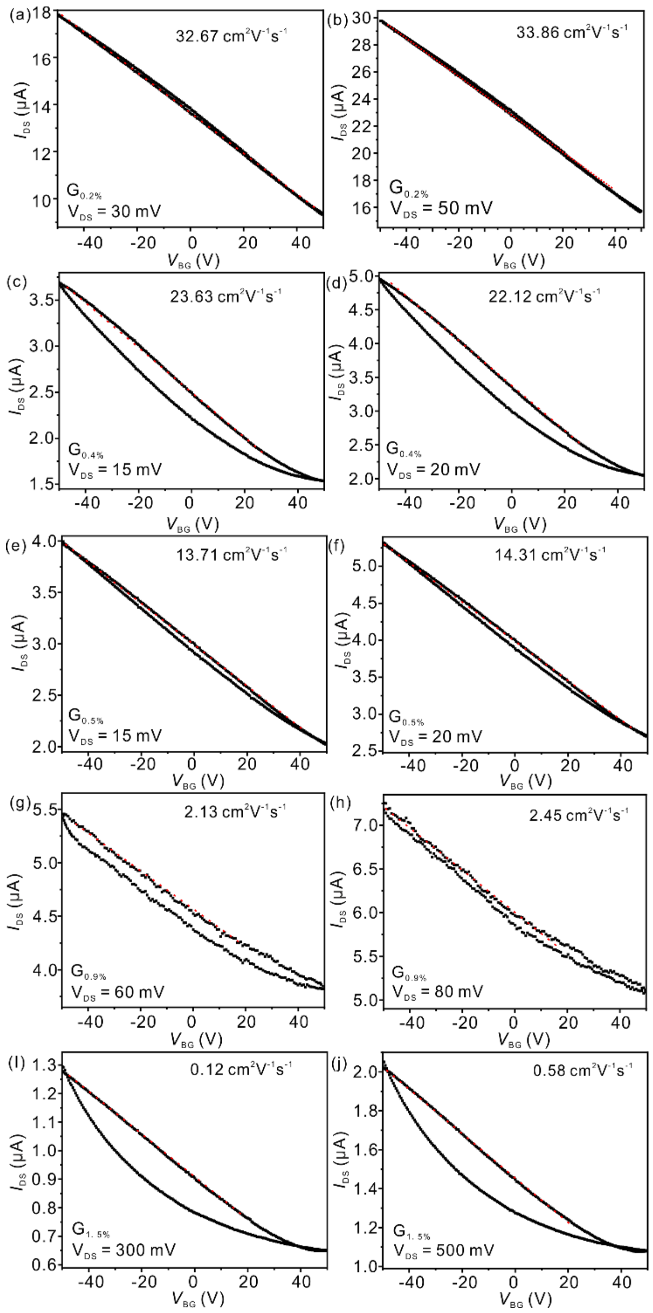

Figure S2. Room-temperature transfer characteristics of graphene transistors with densities of defects of 0%, 0.2%, 0.4%, 0.5%, 0.9% and 1.5%, respectively

Reference

[1] a) L. G. Cançado, A. Jorio, E. H. M. Ferreira, F. Stavale, C. A. Achete, R. B. Capaz, M. V. O. Moutinho, A. Lombardo, T. S. Kulmala and A. C. Ferrari, *Nano Lett.* **2011**, *11*, 3190-3196; b) J. M. Engiert, P. Vecera, K. C. Knirsch, R. A. Schäfer, F. Hauke and A. Hirsch, *ACS Nano* **2013**, *7*, 5472-5482; c) M. M. Lucchese, F. Stavale, E. H. M. Ferreira, C. Vilani, M. V. O. Moutinho, R. B. Capaz, C. A. Achete and A. Jorio, *Carbon* **2010**, *48*, 1592-1597; d) A. C. Ferrari and D. M. Basko, *Nat. Nanotechnol.* **2013**, *8*, 235-246; e) L. G. Cançado, M. G. da Silva, E. H. M. Ferreira, F. Hof, K. Kampioti, K. Huang, A. Penicaud, C. A. Achete, R. B. Capaz and A. Jorio, *2D Mater.* **2017**, *4*, 025039-025050.
